# Hydrogen Sulfide as a Potential Alternative for the Treatment of Myocardial Fibrosis

**DOI:** 10.1155/2020/4105382

**Published:** 2020-01-23

**Authors:** Se Chan Kang, Eun-Hwa Sohn, Sung Ryul Lee

**Affiliations:** ^1^Department of Oriental Medicine Biotechnology, College of Life Sciences, Kyung Hee University, Yongin 17104, Republic of Korea; ^2^Department of Herbal Medicine Resource, Kangwon National University, Samcheok 25949, Republic of Korea; ^3^Department of Convergence Biomedical Science, Cardiovascular and Metabolic Disease Center, College of Medicine, Inje University, Busan 47392, Republic of Korea

## Abstract

Harmful, stressful conditions or events in the cardiovascular system result in cellular damage, inflammation, and fibrosis. Currently, there is no targeted therapy for myocardial fibrosis, which is highly associated with a large number of cardiovascular diseases and can lead to fatal heart failure. Hydrogen sulfide (H_2_S) is an endogenous gasotransmitter similar to nitric oxide and carbon monoxide. H_2_S is involved in the suppression of oxidative stress, inflammation, and cellular death in the cardiovascular system. The level of H_2_S in the body can be boosted by stimulating its synthesis or supplying it exogenously with a simple H_2_S donor with a rapid- or slow-releasing mode, an organosulfur compound, or a hybrid with known drugs (e.g., aspirin). Hypertension, myocardial infarction, and inflammation are exaggerated when H_2_S is reduced. In addition, the exogenous delivery of H_2_S mitigates myocardial fibrosis caused by various pathological conditions, such as a myocardial infarct, hypertension, diabetes, or excessive *β*-adrenergic stimulation, via its involvement in a variety of signaling pathways. Numerous experimental findings suggest that H_2_S may work as a potential alternative for the management of myocardial fibrosis. In this review, the antifibrosis role of H_2_S is briefly addressed in order to gain insight into the development of novel strategies for the treatment of myocardial fibrosis.

## 1. Introduction

Although fibrosis is an essential process for the restoration and maintenance of organ integrity after injury or stress via the timely deposition of the extracellular matrix (ECM), the aberrant accumulation of stiff and disorganized ECM progressively disrupts tissue function and can ultimately cause organ failure [1–5]. Myocardial fibrosis is a hallmark feature of heart failure and is associated with hypertension, myocardial infarction (MI), and pathological hypertrophy followed by injury and stress [1, 2]. Systemic responses induced by the decline in systolic function, particularly neurohumoral activation (angiotensin–aldosterone system and *β*-adrenergic nervous system), are associated with the progression of heart failure (HF). Traditional therapies, such as *β*-blockers and renin-angiotensin-aldosterone system (RAAS) inhibitors, have been found to have beneficial effects in patients with cardiac fibrosis in clinical trials [6, 7]. However, these conventional drugs do not aim at directly curing myocardial fibrosis but rather aim at alleviating the underlying cardiac dysfunction mechanisms indirectly [6]. Therefore, great effort is currently being devoted to research on the development of therapeutic interventions for decreasing the high morbidity and mortality associated with myocardial fibrosis, particularly on the identification and modulation of its core mechanisms [1–4].

Despite its previous characterization as a toxic gas with a rotten egg smell, H_2_S is beginning to be associated with a growing family of gasotransmitters, with properties similar to nitric oxide (NO) and carbon monoxide (CO) [8–10]. As a gasotransmitter, H_2_S is involved in both the physiology and pathophysiology of the nervous, cardiovascular, and gastrointestinal systems via its antioxidant, anti-inflammatory [11], antinociceptive, antihypertensive, neuromodulative, and cytoprotective effects [9, 12–14]. The modulation of signals involved in myocardial fibrosis, and thereby the attenuation of pathological fibrosis, is an area of intense scientific interest due to its evident therapeutic implications for the treatment of HF [15]. Reduced levels of H_2_S have been identified in patients with ischemia [16], diabetes [17, 18], high-fat diet-induced cardiomyopathy [19], hypertension [20], and heavy metal detoxifications, such as nickel detoxification [21].

The role of an exogenously delivered H_2_S in antifibrosis has been identified in a variety of experimental settings (Table 1). In this review, myocardial fibrosis and the potential antifibrosis effects of H_2_S are outlined. H_2_S is not the sole gasotransmitter in the body and can interact with other gasotransmitters including NO and CO. In addition to direct chemical crosstalk, NO, CO, and H_2_S compete in heme- or metal-containing proteins and at the posttranslational modification sites of proteins [9]. Thus, various types of crosstalk between CO, H_2_S, and NO in the cardiovascular system exist [9]. For example, nitrosopersulfide, polysulfides, and dinitrososulfite can be formed by the interaction of NO and H_2_S. These anionic intermediates modulate the bioavailability of NO/HNO or H_2_S/sulfane sulfur and are thus responsible for distinct physiological consequences [22]. Although bioactive intermediates that form interactions with each other are an emerging research field, the modulatory role of H_2_S intermediates in myocardial fibrosis is beyond our current review.

## 2. Hydrogen Sulfide

### 2.1. Synthesis of H_2_S

**Figure 1:**
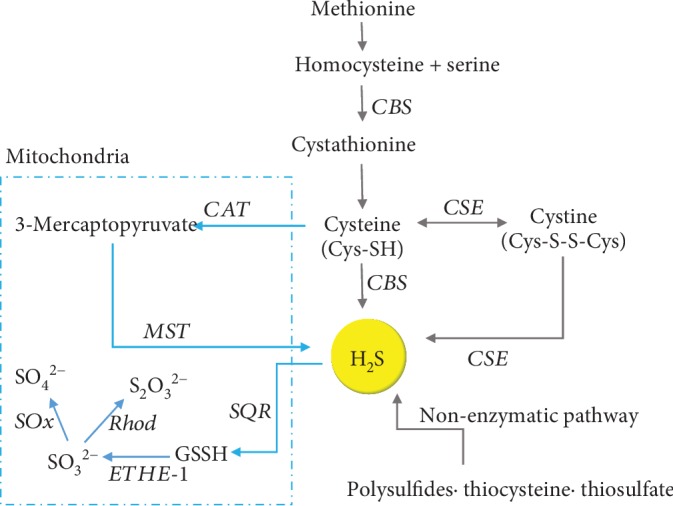
Endogenous H_2_S production. Abbreviations: CAT: cysteine aminotransferase; CBS: cystathionine-*β*-synthase; CSE: cystathionine-*γ*-lyase; ETHE-1: persulfide dioxygenase; GSSH: oxidized glutathione; MST: 3-mercaptopyruvate sulfurtransferase; Rhod: rhodanase; SOx: sulfur dioxygenase; SQR: sulfide quinone oxidoreductase.

H_2_S is the simplest thiol, which are sulfur analogs of alcohol (R-SH); is associated with the smell of rotten eggs; and has a high redox potential [23]. As depicted in Figure 1, H_2_S is endogenously synthesized from L-cysteine or L-homocysteine via cystathionine *β*-synthase (CBS) and cystathionine *γ*-lyase (CSE), which are pyridoxal 5′-phosphate-dependent cytosolic enzymes in the transsulfuration pathway [24]. CSE is involved in the cardiovascular system, especially in myocardial cells [25], vascular smooth muscle cells [26, 27], and endothelial cells [28], whereas CBS is predominantly found in the nervous system [29]. In the mitochondria, cysteine aminotransferase (CAT) catalyzes L-cysteine and glutamate to 3-mercaptopyruvate and *α*-ketoglutarate. Then, 3-mercaptopyruvate is metabolized to pyruvate and H_2_S via 3-mercaptopyruvate sulfurtransferase (3-MST) [23]. Nonenzymatically, H_2_S can also be released from preexisting intracellular sulfur stores (sulfane sulfur) through the activities of reducing agents [24, 30]. For example, the production of H_2_S from sulfur-containing amino acids (e.g., cysteine) via iron and vitamin B_6_ under physiological conditions has been found in red blood cells and tissues [31]. However, the exact biological roles of this nonenzymatic production of H_2_S have not yet been established.

### 2.2. Exogenous H_2_S

H_2_S can be inhaled directly, and the regulated inhalation of H_2_S is an effective method for the control of hemorrhages in preclinical studies [32]. Although the inhalation of H_2_S gas produces few byproducts, controlling its dosage and handling the specialized equipment needed for its delivery is difficult. There are a number of compounds that have been synthesized specifically to deliver therapeutic H_2_S to tissues [9, 23, 33], including inorganic sulfide salts (e.g., NaHS), synthetic organic compounds with a slow H_2_S-releasing mode, conventional drug molecules coupled with an H_2_S-donating group, cysteine analogs, nucleoside phosphorothioates, and plant-derived polysulfides (Table 1).

### 2.3. Modulation of H_2_S Level

The bioavailability of H_2_S inside the cell is primarily regulated by H_2_S-synthesizing enzymes (CSE, CBS, or 3-MST) and H_2_S-oxidizing enzymes located in the mitochondria (e.g., sulfide quinone reductase, persulfide dioxygenase, and thiosulfate sulfurtransferase) [9]. Cysteine and its derivatives can be used to boost H_2_S synthesis [33]. MicroRNA (miRNA) controls gene expression at the posttranscriptional level [34] and is one of the main factors involved in the upregulation of CSE expression [16]. Interestingly, currently used drugs, including angiotensin-converting enzyme (ACE) inhibitors (e.g., ramipril) [35], statins [36], calcium channel antagonists (e.g., amlodipine) [37], digoxin [38], vitamin D_3_ [39], aspirin [40], metformin [40], and others [23], may increase the production of H_2_S. For example, statins can increase H_2_S synthesis via Akt-mediated upregulation of CSE [36] or suppress H_2_S degradation by decreasing the concentration of coenzyme Q, which is a sulfide quinone reductase cofactor [41]. It is worth noting that either exogenously supplied or endogenously produced H_2_S can be stored in the body in the form of bound sulfane, which is a reductant labile sulfur (e.g., persulfide (R-S-S-SH), polysulfide (RSS_n_SR), and protein-associated sulfur, among others) [42]. With regard to the dietary supplementation of H_2_S, garlic and garlic-derived organic polysulfides, such as diallyl trisulfide (DATS) and diallyl disulfide (DADS), behave as H_2_S donors with the aid of a biological thiol (e.g., glutathione), maintained via pentose phosphate pathway-mediated NADPH production [43].

### 2.4. Functional Roles of H_2_S in the Biological System

H_2_S displays antioxidant effects through the direct quenching of reactive oxygen species (ROS) via a hydrosulfide anion (HS-), which is a powerful one-electron chemical reductant that is dissociated from H_2_S in physiological fluid [12]. H_2_S derivatives such as nitrosopersulfide, polysulfides, and dinitrososulfite may also be involved in redox switching in biological systems by generating redox congeners like nitroxyl, nitrous oxide, and sulfane sulfur [22]. NaHS may indirectly suppress ROS production through the H_2_S-mediated activation of a copper/zinc superoxide [44, 45]. In addition, H_2_S induces the suppression of oxidative stress through the activation of Nrf2 (transcription factor nuclear factor (erythroid-derived 2)-like 2) and NAD-dependent deacetylase sirtuin (SIRT)-3, resulting in increased expression of other antioxidant enzymes and proteins (e.g., GSH and thioredoxin-1) [46, 47]. The low concentration of H_2_S may cause oxidative stress, resulting in the depletion of tetrahydrobiopterin, which determines the levels of endothelial nitric oxide synthase (eNOS) activity [48]. As latent matrix metalloproteinases (MMPs) can be activated by oxidative stress [49], the antioxidant capacity of H_2_S may be involved, at least in part, in the suppression of MMP activation.

H_2_S is able to modulate the functions of proteins containing prosthetic metal complexes in acceptor proteins due to its high reactivity with metal ions [50, 51]. For example, polysulfides bind to inactive ferric indoleamine 2,3-dioxygenase (IDO1), which strongly suppresses the immune response, thereby reducing it to its oxygen-binding ferrous state, thus activating IDO1 to maximal turnover [52]. As such, H_2_S is able to elicit an anti-inflammatory response through the activation of IDO1. H_2_S can lead to protein S-sulfhydration (sulfuration or persulfidation) by covalently converting the -SH group of cysteine into an -SSH group in the protein [53], thereby altering the activities of various enzymes, including that of F_1_F_0_-ATPase [54], the ATP-sensitive potassium (K_ATP_) channel [55], and the phosphatase and tensin homolog (PTEN) [56]. In addition, protein sulfhydration changes the localization and stability of proteins inside cells and increases the resistance of proteins to oxidative stresses [54, 55]. H_2_S can activate soluble guanylyl cyclase (sGC) via direct heme binding [57] or by the inhibition of the cGMP phosphodiesterase (PDE) activity [57], resulting in the activation of cyclic GMP (cGMP)–protein kinase G (PKG) pathways.

The bioavailability of H_2_S may play an important role in the integrated stress response, that is, in coping with changes to the cellular environment [58, 59]. H_2_S transiently increases the phosphorylation of eukaryotic initiation factor 2 (eIF2*α*) via the inhibition of protein phosphatase-1 (PP1c) via H_2_S-driven persulfidation [59], thereby inducing a transient adaptive reprogramming of global mRNA translation independent of upstream kinases [59]. As an epigenetic modulator, H_2_S can modify the expression of Brahma-related gene 1 (Brg1) at the promoter region, thus suppressing the transcriptional activity of the ATP-dependent chromatin remodeling complex [60]. This suppressive activity of H_2_S in the expression of Brg1 contributes to the inhibition of vascular smooth muscle cell proliferation [60]. H_2_S may be involved in the decrease of the lysine acetylation of enzymes involved in fatty acid *β*-oxidation and glucose oxidation in diabetic conditions, thereby exerting a beneficial effect on cardiac energy substrate utilization by favoring a switch from fatty acid oxidation to glucose oxidation [61].

Mitochondrial damage associated with cardiovascular pathological stimuli, including oxidative stress, the over-activation of the renin-angiotensin-aldosterone and adrenergic systems, and the dysfunction of growth hormones, plays a central role in the loss of ischemic, and even nonischemic, cardiomyocytes [62, 63]. The levels of mitochondrial DNA (mtDNA) content are dramatically reduced in CSE gene-knockout mice; however, this reduction can be reversed via the exogenous delivery of H_2_S [64]. H_2_S can induce the replication of mtDNA and mitochondrial biogenesis by suppressing the methylation of mitochondrial transcription factor A (TFAM) [64]. In a different way, H_2_S may be involved in the stimulation of cardiac mitochondrial biogenesis through the activation of the 5′ AMP-activated protein kinase (AMPK)-peroxisome proliferator-activated receptor gamma coactivator 1-alpha (PGC1*α*) pathway [65]. The sulfhydration of AMPK and protein phosphatase 2A (PP2A), which leads to the activation of AMPK and the inhibition of PP2A, respectively, has been suggested as a mechanism that may be involved in the H_2_S-mediated stimulation of mitochondrial biogenesis under nonstressed conditions [65].

## 3. Myocardial Fibrosis and Antifibrosis Potential of H_2_S

### 3.1. Myocardial Fibrosis

**Figure 2:**
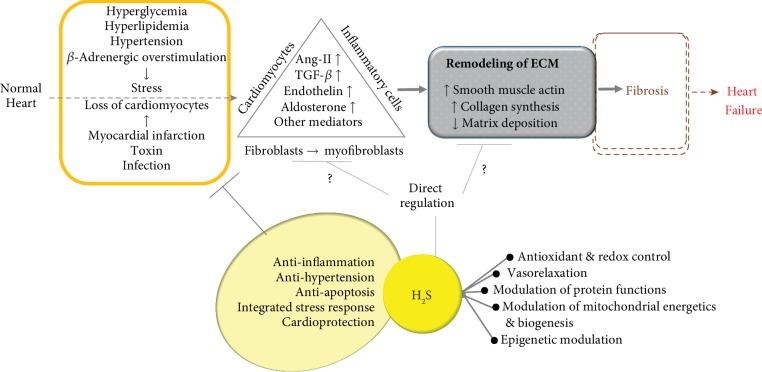
Simplified process of myocardial fibrosis and possible antifibrosis roles of H_2_S. Abbreviations: Ang-II: angiotensin-II; ECM: extracellular matrix; H_2_S: hydrogen sulfide; TGF-*β*: transforming growth factor-*β*.

The heart is a highly organized structure composed of cardiomyocytes and noncardiomyocytes such as fibroblasts (nonexcitable cells of mesenchymal origin), endothelial cells, and vascular smooth muscle cells [66, 67]. Maladaptive crosstalk between cardiomyocytes and noncardiomyocytes responding to pathological stress may result in myocardial fibrosis, adverse remodeling, and arrhythmogenesis. Myocardial fibrosis is a reparative process involving the restoration of cardiomyocytes from cell death or sustained stress and is involved in maintaining the integrity of the heart, an action exerted mainly by the fibrillar, collagen-rich extracellular matrix (ECM), in the short term [68]. However, reactive fibrosis, such as interstitial and perivascular fibrosis [69], contributes to the progressive architectural remodeling of the heart as a result of the formation and deposition of excess fibrous connective tissue [70]. RAAS, transforming growth factor-*β* (TGF-*β*), and *β*-adrenergic systems are common contributors to cardiac remodeling. These systems are connected to each other in an auto-/paracrine manner as a part of a larger signaling network [71]. During the progression of myocardial fibrosis, various distinct immunological and molecular mechanisms are interconnected via interactions between various cells, including macrophages, myofibroblasts, and matrices [68, 70, 72, 73]. As depicted in Figure 2, the loss of cardiomyocytes driven by various injurious agents and stresses has a detrimental effect on the architecture and function of the heart due to the negligible regenerative capacity of the heart, especially with regard to cardiomyocytes [72, 74]. Inflammatory cells, such as macrophages, appear in damaged regions of the heart and are tasked with removing the necrotic cardiomyocyte debris. TGF-*β* is the best-known fibrogenic growth factor involved in cardiac fibrosis, even though a baseline level of TGF-*β* signaling or an early-responsive increase in TGF-*β* may protect the heart from acute injury [75]. It has been demonstrated that angiotensin-II (Ang-II) is an important mediator of cardiac fibrosis, working with the TGF-*β* in the fibrotic response, due to the coexistence of TGF-*β* receptors and Ang-II receptors in cardiomyocytes, inflammatory cells, and cardiac fibroblasts. TGF-*β*1 triggers the appearance of inflammatory cells and myofibroblasts at the site of injury [75, 76] and stimulates the deposition of ECM, including fibronectin, fibrillar collagen types I and III, and proteoglycans. During this initial stage, in addition to the production of inflammatory cytokines, inflammatory cells secrete Ang-I, which is converted to Ang-II via the action of ACE. Ang-II plays a pivotal role in stimulating TGF-*β* production, prompting the proliferation of circumambient fibroblasts and their transdifferentiation into myofibroblasts. The pool of fibroblasts can be enlarged by the transformation of either circulating bone marrow cells or endothelial/epithelial cells into fibroblasts [66, 77]. During the proliferative phase of cardiac repair, fibroblasts undergo transdifferentiation into contractile myofibroblasts, secreting large amounts of matrix proteins, such as collagens [66]. Then, the scar tissue matures with the formation of a collagen-based matrix [78], where the removal of myofibroblasts is controlled by unknown endogenous stop signals in order to restrain the fibrotic response [78]. However, a clear mechanistic view of phenotype and heterogeneity of cardiac fibroblasts in the process of fibrosis has yet to be fully established [77]. In terms of the underlying molecular mechanisms involved in the progression of fibrosis, several pathways, including the TGF-*β*, JNK/p38, PI3K/AKT, WNT/*β*-catenin, and Ras-Raf- mitogen-activated protein kinase- (MEK-) extracellular signal-activated kinase (ERK) pathways, have been identified [79]. Involved in canonical fibrotic signaling, TGF-*β* induces the nuclear translocation of the complex known as “mothers against decapentaplegic homolog,” or SMAD complex promoting fibrosis. In noncanonical signaling, TGF-*β* signaling induces SMAD-independent pathways, including the PI3K/AKT and mitogen-activated protein kinase (MAPK) pathways, nuclear factor kappa light chain enhancer of activated B cell (NF-*κβ*), RHO/RAC1, and CDC42 [75]. Interestingly, it has been suggested that, if supplied in a timely manner, H_2_S can suppress TGF-*β*1-induced transdifferentiation from fibroblasts to myofibroblasts via the inhibition of SMAD3 activation in human fibroblast cells [80].

### 3.2. Antifibrosis Potential of H_2_S

#### 3.2.1. Myocardial Infarction

Extensive necrosis of cardiomyocytes in infarcted hearts not only triggers a strong inflammatory response but also induces interstitial and perivascular fibrosis due to geometrical, biomechanical, and biochemical changes in the uninjured ventricular wall [69]. During cardiac injury and hypertrophic remodeling, the production of inflammatory signaling molecules, such as tumor necrosis factor-*α* (TNF-*α*), interleukin (IL)-1*β*, and IL-6, can contribute to hypertrophic and fibrotic responses. Interestingly, ischemia causes a significant reduction in the levels of H_2_S associated with decreased expression of CSE, which is an H_2_S-synthesizing enzyme under the control of the miRNA-30 family [16]. Moreover, it has been suggested that reduced plasma H_2_S levels are correlated with the severity of coronary heart disease [81]. Similar to the cardioprotective role of NO [82–84], various signaling pathways from different types of exogenous H_2_S may be involved in the suppression of MI-associated fibrosis (Table 1). These pathways include GSK-3*β*/*β*-catenin [85], cGMP-PKG [86], Nrf2 [87–89], miRNA signaling pathways [16, 90, 91], and the protection of mitochondria [92–95]. Although postconditioning only exerts cardioprotection in young hearts, exogenous H_2_S restores postconditioning benefits by upregulating autophagy via the activation of the AMPK/mammalian target of rapamycin (mTOR) pathway in the aged hearts and cardiomyocytes [96]. It is unclear whether the signaling pathways identified share common contributors derived from H_2_S, or whether this is simply the result of experimental settings targeting different signaling pathways. Therefore, the identification of a unique contributor of H_2_S involved in the suppression of MI-mediated myocardial fibrosis is necessary.

#### 3.2.2. Hypertension

Hypertension increases oxidative stress, vascular inflammation, and vascular remodeling, such as in the case of fibrosis [97]. The antihypertensive effects of H_2_S, associated with its modulation of various levels of channel activity and cGMP-PKG pathways, may contribute to the suppression of fibrosis caused by hypertension [98–101]. As presented in Table 1, H_2_S supplementation under hypertensive conditions may suppress myocardial fibrosis via the modulation of several different signaling pathways. It is worth noting that H_2_S can inhibit ACE via the binding of zinc ions to the active center of ACE [102]. It has been postulated that the H_2_S-mediated suppression of ACE may indirectly contribute to vasorelaxation and the suppression of the Ang-II-mediated transition of fibrosis. Alternatively, the suppression of inflammation [103] and the reduction of cardiomyocyte death from oxidative stress [104], as well as the activation of eNOS/NO pathway [105], are likely to have antifibrosis roles with regard to H_2_S under hypertensive conditions. Interestingly, it has been noted that local delivery of H_2_S can lower systemic blood pressure. For example, the intra-cerebroventricular (ICV) infusion of NaHS in both spontaneous and Ang-II-induced hypertensive rat models was found to decrease the mean arterial blood pressure and heart rate during ICV infusions [106]. Moreover, H_2_S secreted from periadventitial adipose tissue has been previously found to contribute to blood pressure homeostasis [107].

#### 3.2.3. Diabetes

The metabolic environment of diabetes, including hyperglycemia, hyperlipidemia, and oxidative stress, causes cardiomyocyte cell death. The early stages of diabetic remodeling of the heart are usually asymptomatic, such that myocardial changes mostly occur at the molecular level. In the middle stage of remodeling, progressive cardiomyocyte hypertrophy and myocardial fibrosis result in impaired ejection fraction [108]. In patients with diabetes, as well as in streptozotocin- (STZ-) treated rats, lowered circulating levels of H_2_S due to the downregulated expression of H_2_S-synthesizing enzymes have been frequently found [109–111]. As depicted in Table 1, several underlying mechanisms of H_2_S involve the suppression of myocardial fibrosis in diabetic rats via (1) the suppression of the TGF-*β*1/SMAD3 pathway [110, 112, 113] and canonical Wnt pathway [114], (2) the suppression of endoplasmic reticulum stress [19, 115], (3) the downregulation of the JAK/STAT signaling pathway [110], and (4) the regulation of autophagy [112, 116]. Although it has not yet been clearly elucidated, there is a possibility that H_2_S may be involved in the modulation of ECM remodeling via miRNA or other transcription machinery affecting the expression of ECM-processing enzymes in diabetes. For example, H_2_S has been found to attenuate fibrotic changes in diabetic kidneys via the downregulation of miRNA-194, which plays an important role in the modulation of proteins involved in collagen realignment [117].

#### 3.2.4. Neurohormonal Overstimulation

The activation of the *β*-adrenergic nervous system and RAAS has been commonly found in fibrotic HF patients, and *β*-blockers and RAAS inhibitors have been suggested as a first-line treatment to correct the underlying cardiac dysfunction and reduce morbidity [7, 118]. The overstimulation of *β*-adrenoceptor may result in the impairment of the negative modulation of H_2_S on the *β*-adrenoceptor system, resulting in a calcium overload, leading to the impairment of cardiac contractility and, ultimately, to cardiomyocyte death [119]. Exogenous H_2_S supplementation inhibits isoprenaline- (ISO-) induced cardiac hypertrophy depending on SIRT3, which is predominantly localized in the mitochondria, and may be associated with antioxidant properties [120]. Other signaling pathways, including reducing NADPH oxidase [121] or S-sulfhydration of Ca^2+^/calmodulin-dependent protein kinase II [122], have been associated with the antifibrosis role of H_2_S under conditions of *β*-adrenoceptor overstimulation. Mast cells will infiltrate into the heart at the site of inflammation and serve as a local source of renin in cardiovascular tissues. H_2_S may benefit from the action of renin secreted from mast cells [123]. In view of hormone-associated myocardial fibrosis, the excessive generation of thyroxine from thyroid induces thyrotoxicosis and affects the cardiovascular system, resulting in the symptoms of hypertension, arrhythmia, and cardiac hypertrophy [124]. Under conditions of excessive thyroxine, H_2_S may bolster rat myocardial fibrosis through the activation of autophagy mediated by the PI3K/AKT signaling pathway and via the downregulation of miRNA-21, miRNA-34a, and miRNA-214 expression [125].

## 4. Summary and Perspectives

Currently, strategies for the treatment of established HF are focused on relieving the symptoms and signs of HF, such as treating edema, preventing hospital admission, and improving survival [6]. Myocardial fibrosis determines the clinical course of heart dysfunction and can eventually lead to heart failure. A substantial amount of research has been dedicated to the identification of HF target(s) to improve the diagnosis and treatment of fibrotic pathways with organ specificity. Myocardial fibrosis has many steps and usually involves several pathways. Complex networks of molecular signaling, including GSK-3*β*, *β*-catenin, and TGF-*β*1/SMAD3, have been implicated in the initiation, progression, and regression of myocardial fibrosis [1–5]. The targeting of collagen fibrillogenesis should be performed with caution as collagen turnover is a common process in most tissues whose effects can be detrimental [150]. Although TGF-*β*1 is a central profibrogenic cytokine and a critical contributor during myocardial fibrosis, treatment with TGF-*β* antibody has been found to result in an increased mortality rate and poor MI-associated ventricular remodeling in a mouse model [151]. Although SMAD3 and TNF-*α* signaling play a fundamental role in fibrosis progression, the targeting of SMAD3 and TNF-*α* antagonism has not yet been found to provide a successful antifibrosis outcome [151]. Based on the important role of Ang-II in the initiation of myocardial fibrosis, the antagonism of the angiotensin pathway via ACE inhibitors and angiotensin receptor antagonists is considered to be a useful approach for the management of fibrotic diseases. Recently, AMPK*α* activators (e.g., metformin) have been found to be a promising therapeutic target for fibrosis [152]. Myocardial fibrosis is not caused by a single profibrotic pathway but is rather associated with the activation of several profibrotic pathways, including immunological and molecular mechanisms [70]. It is also worth noting that a combined antifibrotic strategy, including inflammatory mediators, profibrotic cytokines, and epigenetic and cell and/or tissue intrinsic changes, has been suggested as a possible method for the successful treatment of myocardial fibrosis [7, 70].

As briefly addressed in this review, H_2_S possesses antioxidant capacities and modulates various signaling pathways, including the activation of cGMP-PKG pathways, the posttranslational modification of proteins, metal-binding (including heme), and mitochondrial respiratory control [9]. In addition, H_2_S may serve as a fine-tuner of mitochondrial homeostasis and the autophagic process in the physiology and pathophysiology of the cardiovascular system [153]. Moreover, H_2_S is involved in antiapoptosis of cardiomyocytes, anti-inflammation, antihypertension, and other beneficial cardiovascular processes [154, 155]. As a timely response to energy stress, autophagy is a bulk degradation/recycling system that is tightly controlled by the homeostatic pathway in the cardiovascular system [153, 156]. Despite the existence of conflicting opinions on the beneficial and harmful effects of autophagy, disturbances in the autophagic process have been found in various forms of HF, including age-related cardiomyopathies [156]. H_2_S may be involved in the regulation of autophagy by either suppressing or enhancing the signaling pathways that contribute to the attenuation of myocardial fibrosis, as reviewed in a previous paper [156, 157]. Although it is still currently under investigation, numerous findings have demonstrated that H_2_S may be involved in the suppression of myocardial fibrosis caused by (1) myocardial infarction, (2) hypertension, (3) STZ-induced diabetes, and (4) the overstimulation of neurohormonal routes (Table 1). The signaling pathways mediated by H_2_S may converge on the suppression of myocardial fibrosis that occurs as a result of various stresses, as shown in Figure 2 and Table 1. It is unclear whether target pathways modulated by the action of H_2_S work independently of each other; however, it is most important to determine whether they allow for the merging of multiple pathways into a single antifibrosis signaling cascade. Versatile mechanisms and signaling pathways triggered by H_2_S have already been identified, as briefly shown in this review. In this context, it appears that H_2_S is emerging as a new type of myocardial fibrosis suppressor. However, it is necessary to identify the molecular target or specific signaling pathway that is under the control of H_2_S in a direct and specific manner during myocardial fibrosis. It remains to be clearly established whether H_2_S can directly control the cells involved in fibrosis (e.g., cardiomyocytes, fibroblasts, and inflammatory cells) and ECM deposition.

The advances being made in H_2_S biology are a promising tool for the future development of medicines for the treatment of myocardial fibrosis based on H_2_S, as well as multitarget molecules able to release H_2_S [158]. There is currently a lack of fibrosis-specific biomarkers that can be used to determine the stage and grade of myocardial fibrosis, as well as for the identification of patients who may benefit from a specific type of therapy. In addition to the development of new techniques for evaluating the stage and/or severity of myocardial fibrosis [159], a new strategy for reversing preexisting fibrosis using H_2_S could be a valuable approach. Moreover, the potential of H_2_S in preventing or repairing cardiomyocyte loss via the stimulation of cardiac stem cells or transdifferentiation from noncardiomyocytes to cardiomyocytes needs to be critically evaluated in future studies [160]. It is worth mentioning that H_2_S can have serious and toxic effects at high concentrations or high release rates, including sudden unconsciousness and death [14, 161]. Therefore, the optimal concentration or dose of H_2_S for the desired antifibrosis effect needs to be critically examined. Additionally, for the therapeutic potential of H_2_S, pharmacological agents that generate or release H_2_S need to be adequately harnessed for the delivery of physiologically relevant concentrations in a safe manner. Considering that myocardial fibrosis is a long-term consequence of heart disease, the study of dietary supplements that are able to supply H_2_S safely or boost H_2_S synthesis is needed for the management of myocardial fibrosis. The long-term consequences and clinical benefits of H_2_S against myocardial fibrosis should also be investigated in the future. In addition, the study of the H_2_S-mediated the study of the H_2_S-mediated reversal of myocardial fibrosis could prove to be advantageous in clinical studies.

## Figures and Tables

**Table 1:** H_2_S donors and their involvement in myocardial fibrosis.

H_2_S donor (characteristics)	Fibrosis model	Suggested mode of action
Sodium thiosulfate (Na_2_O_3_S) (Hydrophilic, fast H_2_S release)	Hypertension	Antihypertensive activity [99] ↓Oxidative stress and ↑endogenous H_2_S production [126]

Sodium hydrosulfide (NaHS) (Hydrophilic, fast H_2_S release)	Diabetes (type I)	↓Canonical Wnt pathway-TGF-*β*1/SMAD3 pathway [114] ↑PKC-ERK1/2MAPK signaling pathway [127] ↓ER stress [115] ↓Autophagy via the upregulation of the PI3K/AKT1 pathway [116]
	Diabetes (type II)	↓Cardiac muscle degradation via sulfhydration of MuRF1 [128]
	Hypertension	↓Inflammatory response [103] ↓Extracellular matrix accumulation and ↑vascular density [129] ↓Nox4-ROS-ERK1/2 pathway and ↑HO-1 expression [130] ↑Akt/eNOS/NO pathway [105] ↓Nitrotyrosine, ↓MMP-2 and MMP-9, and ↑TIMP-1 and TIMP-3 [131] ↑Opening of K_ATP_ channels and ↓oxidative stress [132]
	Myocardial infarction	↓Oxidative stress [133] ↓iNOS and ↑HO-1 expression [134] ↓mPTP opening in the aging cardiomyocytes [92] ↑Glycogen synthase kinase-3*β*/*β*-catenin pathway [85] ↑cGMP-dependent PKG/phospholamban pathway [135] ↑Angiogenesis [136] ↑Autophagy in the aged hearts [96]
	Metal toxicity (e.g., nickel)	↓Sp1 transactivation and ↓TGF-*β*1/SMAD1 pathway [21]

Sodium sulfide (Na_2_S) (Hydrophilic, H_2_S release for ~520 min *in vivo*; ~5 min *in vitro*)	Hypertension	↑Vasorelaxation [100, 101] ↑Trx1 [137]
	Myocardial infarction	↑Mitochondrial function [95] ↑Nrf2 signaling pathway [87] ↑miR-21 [91]

GYY4137 (Hydrolysis-triggered H_2_S release; slow-release and 3–4 h lasting)	Diabetes (type I)	↓FoxO1 pathway [138] ↑Autophagy via the AMPK/mTOR pathway [112] ↑Nrf2 pathway mediated by sulfhydration of Keap1 [88]
	Myocardial infarction	↑PKG I [139] ↓Oxidative stress and ↓apoptosis [140, 141]

AP67/AP72 (Slow-releasing H_2_S donors)	Atherosclerosis	↓Calcification effects in heart valves [142]

AP39 (Mitochondria-targeted H_2_S)	Myocardial infarction	↓mPTP opening [93, 94] ↓Cardiomyocyte death and ↓inflammatory response [143]
SG-1002 (Orally active)	Hypertension	↑VEGF-Akt-eNOS-NO-cGMP signaling pathway [144]
	Myocardial dysfunction	↑Adiponectin-AMPK signaling and ↓ER stress [19]
		↑NO bioavailability [145]

Diallyl disulfide (DAD) [146] (Organosulfur compound; insoluble in water; slow H_2_S donor [146])	Myocardial infarction	↓ER stress via SIRT1 [147] ↑AMPK-mediated AKT/GSK-3*β*/HIF-1*α* activation [148]

Diallyl trisulfide (DAT) [146] (Organosulfur compound; fast H_2_S donor)	Hyperglycemia	↑PI3K/Akt/Nrf2 pathway [89] ↓JNK/NF-*κβ* pathway [149]

ADT-OH (H_2_S-aspirin hybrid molecule)	Myocardial infarction	↑Autophagic flux via activating AMPK [94]

ZYZ-803 (H_2_S-NO hybrid molecule)	Adrenergic overload	↑Activation of VEGF/cGMP pathway [86]

ZYZ-802 (S-Propargyl-cysteine; cysteine derivatives)	Hypertension	↓Oxidative stress and ↓cardiomyocyte death [104]
	Myocardial infarction	↓miRNA-30 family [16]

Abbreviations: AMPK: AMP-activated protein kinase; Ang-II: angiotensin-II; cGMP: cyclic guanosine monophosphate; ECM: extracellular matrix; eNOS: endothelial nitric oxide synthase; ER: endoplasmic reticulum; ERK: extracellular-signal-regulated kinase; FoxO1: transcription factor Forkhead 1; GSK-3*β*: glycogen synthase kinase-3*β*; H_2_S: hydrogen sulfide; HIF-1*α*: hypoxia-inducible factor-1*α*; HO-1: heme oxygenase-1; iNOS: inducible nitric oxide synthase; JNK: c-Jun N-terminal kinase; Keap1: Kelch-like ECH-associated protein 1; MAPK: mitogen-activated protein kinase; MI: myocardial infarction; miRNA: microRNA; MMP: matrix metalloproteinase; mPTP: mitochondrial permeability transition pore; NOX4: NADPH oxidase 4; MuRF1: muscle RING-finger protein-1; NF-*κβ*: nuclear factor kappa light chain enhancer of activated B cell; Nrf2: nuclear factor E2-related factor 2; PI3K: phosphoinositide 3-kinases; PKC: protein kinase C; PKG: protein kinase G; SIRT1: sirtuin 1; SMAD: mothers against decapentaplegic homolog; Sp1: specificity protein 1; TGF-*β*: transforming growth factor-*β*; TIMP: tissue inhibitor of metalloproteinase; Trx1: thioredoxin 1; VEGF: vascular endothelial growth factor. ↓ and ↑ denote inhibition or suppression and increase or activation, respectively.
